# Spatio-temporal dataset of energy infrastructure attacks in Nigeria (2009–2025)

**DOI:** 10.1016/j.dib.2026.112786

**Published:** 2026-04-16

**Authors:** Haruna Inuwa, Alycia Leonard, Stephanie Hirmer

**Affiliations:** Department of Engineering Science, University of Oxford, Parks Road, Oxford, OX1 3PJ, UK

**Keywords:** Energy infrastructure, Oil and Gas, Nigeria, Energy security, Conflict resilience

## Abstract

This dataset compiles attacks on energy infrastructure in Nigeria between 2009 and 2025. It integrates geospatially attributed incident-level data with aggregated estimates of physical and economic losses. The dataset covers deliberate disruptions to oil and gas infrastructure and electricity transmission systems across Nigeria's six geopolitical zones. It consists of two complementary components. The first is a geo-referenced incident data (2011–2025) consisting of 161 recorded attacks, with information on date, location, actor type, targeted asset, and attack method. The second is an annual volume-value dataset (2009–2025) reporting crude oil losses and associated revenue estimates resulting from pipeline vandalism, crude theft, and spill events. Data were compiled from verified open sources, including the Nigeria Security Tracker (Council for Foreign Relations), domestic and international media reports, and industry and regulatory publications. These data were processed using reproducible filtering and classification scripts. Together, these datasets enable quantitative assessment of the physical scale and economic implications of crude oil theft in Nigeria. They also support comparative, temporal, and scenario-based analyses across research, policy, and public-interest applications. All data and supporting files are openly available in a Zenodo repository.

Specifications Table

**Table:** 

Subject	Engineering & Materials science
Specific subject area	Energy infrastructure security, oil theft, and geospatial conflict-related infrastructure attacks in Nigeria
Type of data	Table, Chart, Graph, Figure; Raw, Filtered, Processed, Time-series data; CSV files; Python scripts
Data collection	Incident-level data were compiled from verified open sources, including the Nigeria Security Tracker (Council on Foreign Relations), peer-reviewed literature, media archives, and government reports. Keyword searches combined actor identifiers, infrastructure terms, and regional modifiers. Records were screened using predefined criteria, geocoded with administrative shapefiles, deduplicated through triangulation, and classified by sector and zone. Annual crude loss volumes (2009-2024) were from industry and government reports.
Data source location	Nigeria (six geopolitical zones: North-East, North-West, North-Central, South-East, South-West, South-South); geocoded at state level; archived on Zenodo.
Data accessibility	Repository name: Zenodo Data identification number: DOI: 10.5281/zenodo.15974757 Direct URL to data: https://zenodo.org/records/15974757 and https://github.com/Diamonds10/data_in_brief_infradataset_Nigeria Instructions for accessing these data: Data and code are openly accessible. Download CSV files and scripts directly from Zenodo or the linked GitHub repository. No login required.
Related research article	None

## 1 Value of the Data

• The incident-level attack dataset (2011–2025) supports spatial, sectoral, and temporal analysis of attacks on oil and gas infrastructure and electricity transmission systems across Nigeria’s six geopolitical zones.
• The dataset provides a harmonized time series of annual crude oil theft volumes and associated revenue losses in Nigeria from 2009 to 2025, enabling transparent comparison of physical and economic impacts across political, security, and market conditions.
• Researchers, policymakers, and regulators—including institutions such as the Nigerian Upstream Petroleum Regulatory Commission (NUPRC), Nigerian National Petroleum Company Limited (NNPCL), Transmission Company of Nigeria (TCN), and international organizations such as the World Bank Group and International Energy Agency (IEA)—can use these estimates to assess fiscal exposure and prioritize infrastructure protection and investment strategies.
• Journalists, investigative media platforms, and civil society organizations—including transparency and accountability groups such as BudgIT and Nigeria Extractive Industries Transparency Initiative (NEITI) may use the data to enhance public reporting and improve transparency regarding the scale, variability, and uncertainty of crude oil theft and energy infrastructure disruptions in Nigeria.
• The dataset can be used within energy systems and risk-modelling frameworks to assess how physical supply losses are reflected in economic outcomes under changing market and policy conditions.

## 2 Background

Attacks on energy infrastructure by non-state actors have become a recurring feature of energy insecurity in politically volatile and resource-dependent nations [1,2]. In Nigeria, incidents involving oil and gas infrastructure and electricity transmission systems have been reported across multiple geopolitical zones over the past two decades [[3], [4], [5]]. Despite their frequency, incident-level data remain fragmented across media reports, regulatory publications, and secondary literature, with limited harmonization for systematic analysis.

This dataset was compiled to consolidate and standardize records of energy infrastructure attacks in Nigeria between 2009 and 2025 within a structured, geo-referenced framework. It integrates incident-level information on date, location, actor category, and associated monetary estimates. Data were assembled from verified open sources and processed using predefined inclusion criteria, geocoding protocols, and cross-source validation procedures [6].

The resulting dataset provides the basis for subsequent spatial and temporal analyses of energy infrastructure insecurity in Nigeria.

## 3 Data Description

The dataset presents spatio-temporal records of attacks on energy infrastructure in Nigeria between 2009 and 2025, supporting descriptive and quantitative assessment of energy insecurity, infrastructure vulnerability, and associated economic and environmental impacts. It captures incidents affecting both oil and gas infrastructure and electricity transmission systems.

The dataset is organized into two complimentary components. The first is an incident-level attack dataset (2011–2025), which describes the distribution of attacks across two sectors—oil and gas, and electricity transmission systems—and across six geopolitical regions. The second is a yearly volume-value loss dataset (2009–2025), which documents crude oil losses and associated revenue estimates resulting from pipeline vandalism, oil theft, and spill events.

Together, these components enable analysis of attack patterns and comparison between physical losses and monetary valuation over time, while accommodating differences in data availability and reporting practices across periods.

### 3.1 Incident-level attack data

**Fig. 1:**
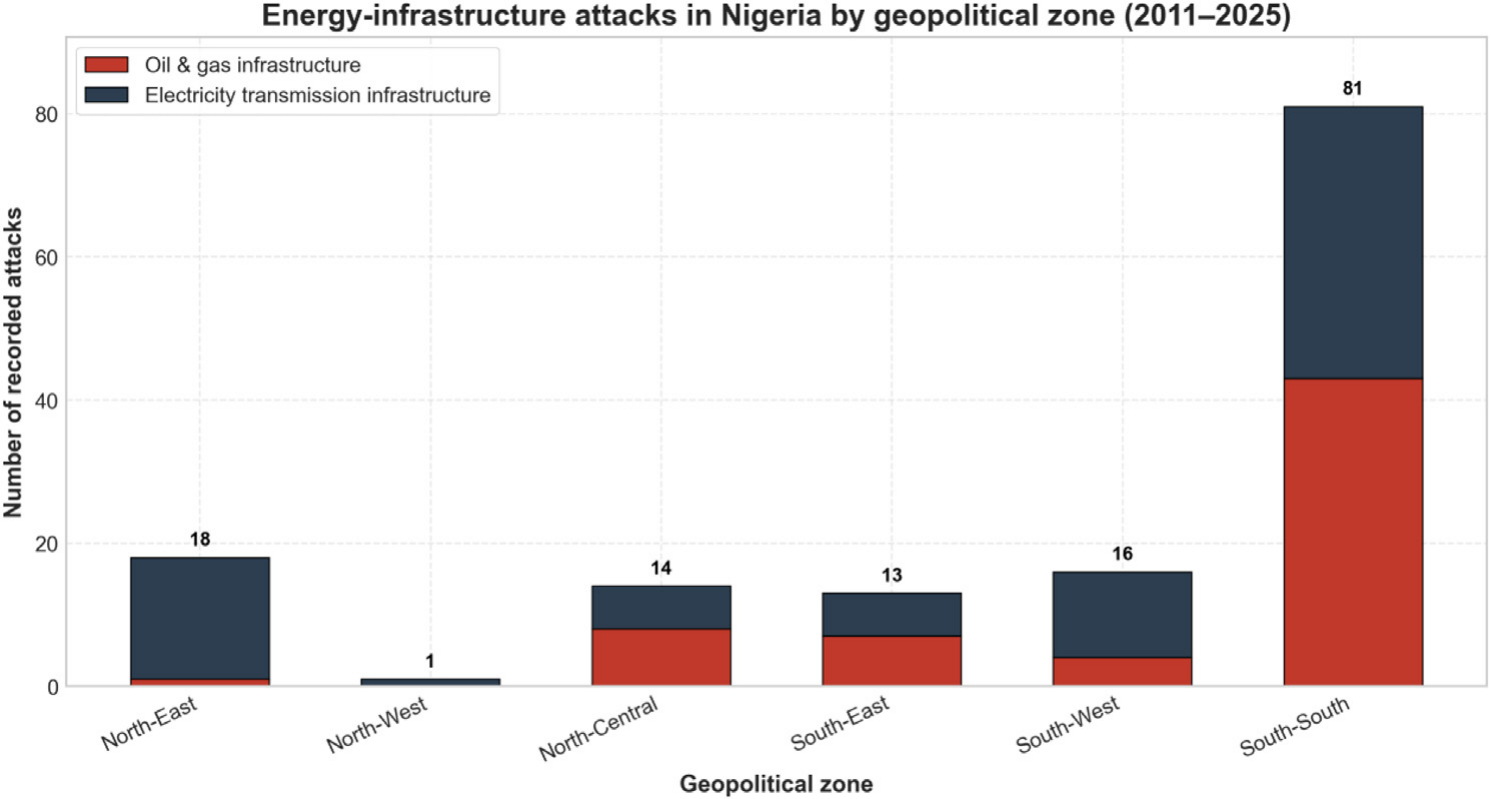
Energy infrastructure attacks in Nigeria in this dataset by geopolitical zone and sector, 2011–2025. Stacked bars show the total number of incidents per geopolitical zone over the 2011–2025 period, with red segments indicating oil and gas attacks, and dark blue segments indicate electricity transmission (power) attacks. The highest number of recorded attacks occurs in the South-South region (81), while the North-West zone has the lowest count (1), indicating substantial change in attack intensity across Nigeria's geopolitical regions. Data from compiled open-source incident reports.

Fig. 1 highlights the spatial distribution of the documented energy infrastructure attacks across Nigeria's six geopolitical zones during 2011–2025.

The underlying data were compiled from open-source incident reports and integratedinto a unified dataset covering oil and gas and electricity transmission systems. Individual incidents were geo-located to the state level and subsquently assigned geopolitical zones using a predefined state-zone concordance. In addition, Fig. 1 presents aggregate incident counts by zone and sector over the study period, providing an overview of the distribution of attacks across different types of energy infrastructure (Table 1).

**Table 1:** Key variables in the incident-level energy infrastructure attack dataset (2011–2025).

Variable	Definition	Sources
Date of incident	Calendar date on which the energy infrastructure attack was reported to have occurred; used to assign year and analysis period.	Compiled open-source records [6]
Location	Reported location of the incident, geolocated at the state level for spatial analysis.	Compiled open-source records [6]
Geopolitical zone	Nigerian geopolitical zone (North-East, North-West, North-Central, South-East, South-West, South-South) assigned using a predefined state–zone concordance.	Author classification based on compiled sources [6]
Infrastructure sector	Category of energy infrastructure targeted, distinguishing between oil and gas assets and electricity transmission systems.	Incident descriptions and classification rules [6]
Non-state actor type	Category of non-state actor reportedly responsible for the incident (e.g., militants, insurgents, bandits, or unknown).	Compiled open-source records [6]
Sectoral distribution	Derived classification enabling aggregation of incidents by infrastructure sector for descriptive analysis.	Derived from compiled dataset [6]
Spatial distribution	Derived classification enabling aggregation of incidents by geopolitical zone for descriptive spatial analysis.	Derived from geocoded records [6]

Derived variables in the dataset are defined through aggregation procedures applied to the underlying incident-level and annual records. Sectoral distribution is derived by classifying and aggregating incidents according to infrastructure type (oil and gas versus electricity transmission systems). Spatial distribution is obtained by aggregating geocoded incidents across Nigeria’s six geopolitical zones.

**Yearly volume-value loss data**

**Fig. 2:**
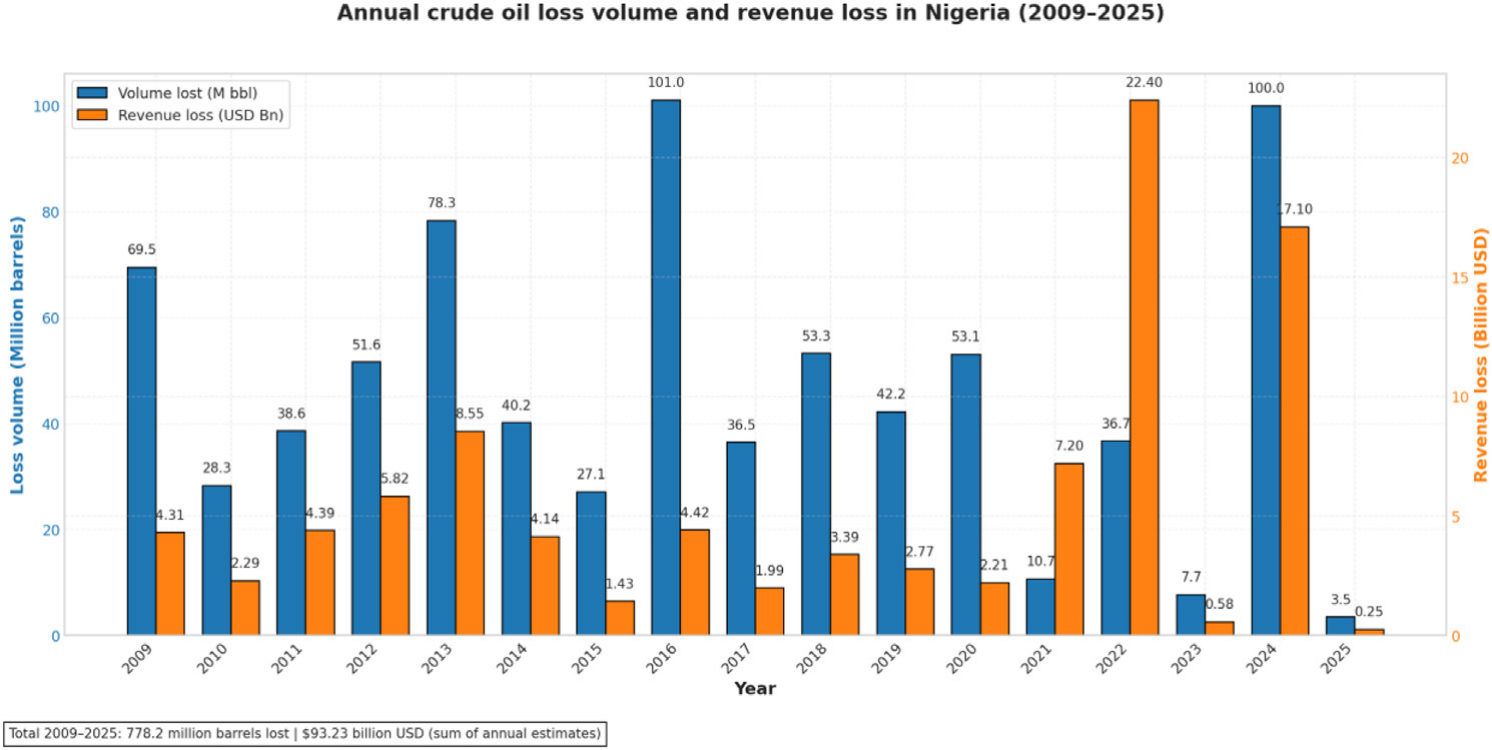
Annual crude oil loss volume and revenue loss in Nigeria (2009–2025). The figure shows estimated annual crude oil losses due to theft, vandalism, and related incidents in Nigeria, expressed as physical losses (million barrels; left axis) and corresponding revenue losses (billion USD; right axis). Annual estimates for 2009–2024 are compiled from industry reports and open-source assessment. The 2025 estimate is based on official Nigerian Upstream Petroleum Regulatory Commission (NUPRC), extrapolated from January–July 2025 data and is linearly extrapolated to represent an indicative annual value. Revenue losses are normalised to USD using year-specific exchange rates. Numeric labels indicate reported annual values. The figure summarises inter-annual variation in reported physical and monetary losses without implying causal relationships.

Fig. 2 summarizes annual estimates of crude losses because of theft and vandalism in Nigeria captured in the dataset between 2009 and 2025. The figure integrates two bar charts (volume and monetary value) derived from the compiled dataset [6].

Estimates for 2009–2024 are based on industry and open-source media reports, whereas the 2025 value reflects an official low estimate reported by the Nigeria Upstream Petroleum Regulatory Commission (NUPRC), extrapolated from data covering January-July 2025 [7]. These data provide a descriptive overview of inter-annual variation in reported crude oil losses and their associated valuation.

Observed variations across years, including the decline in 2023 and the increase in 2024, reflect differences in reporting sources and changes in the number of recorded incidents across years. In particular, the lower values in 2023 coincide with a reduced number of documented attacks, while the higher values in 2024 reflect increased reported incidents and higher reported loss estimates. The 2025 value, derived from partial-year data and subsequently extrapolated, should therefore be interpreted as indicative rather than directly comparable with other years (Table 2).

**Table 2:** Key variables in the annual volume–value crude oil loss dataset (2009–2025).

Variable	Definition	Sources
Annual crude oil loss volume	Estimated annual volume of crude oil lost due to pipeline vandalism, oil theft, and spill events, reported in million barrels for each year between 2009 and 2025.	Compiled open-source records [[6], [8], [9]]
Annual revenue loss	Corresponding economic value of reported crude oil losses, normalised to billion USD using year-specific exchange rates.	Compiled open-source records; World Bank exchange rates [6,10]
Temporal variation	Year-to-year variation in reported crude oil loss volumes and associated revenue estimates, reflecting changes in reported loss magnitudes and prevailing pricing conditions.	Derived from annual loss records [6]

Temporal variation is derived from annual aggregation of crude oil loss volumes and associated revenue estimates, enabling year-on-year comparison of reported losses.

### 3.2 Crude oil losses relative to production

**Fig. 3:**
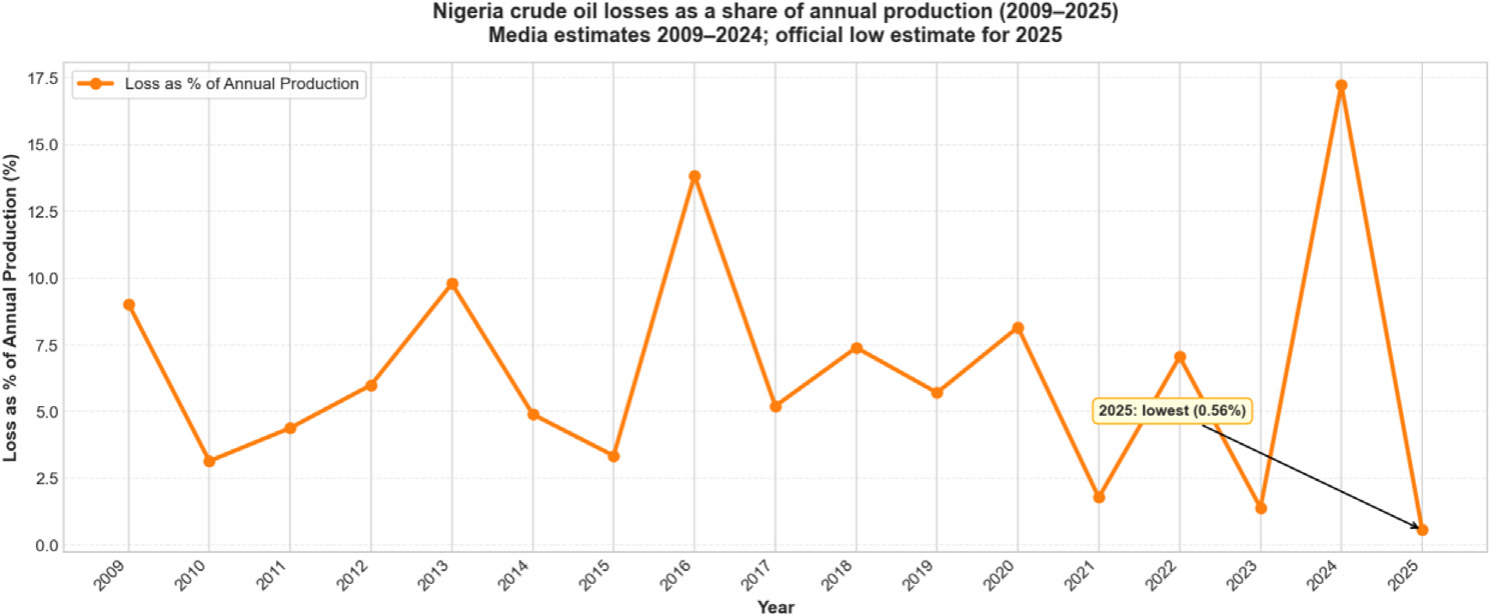
Crude oil losses as a share of annual production in Nigeria (2009–2025). The figure shows estimated crude oil losses expressed as a percentage of total annual national production. Values for 2009–2024 are based on industry and open-source loss estimates combine with reported annual production figures, while the 2025 value reflects an official low estimate reported by the Nigerian upstream Petroleum Regulatory Commission (NUPRC), extrapolated from January-July 2025 data. The figure presents a production-adjusted view of loss intensity over time.

Fig. 3 presents annual crude oil losses expressed as a percentage of total national production in Nigeria between 2009–2025, derived from the volume-value dataset [6]. By expressing losses relative to annual production, the figure enables comparison across years with production levels. This production-adjusted representation highlights changes in loss intensity over time, independent of fluctuations in overall output.

## 4 Experimental Design, Materials and Methods

A structured approach was taken to compile, classify, and describe incidents of energy infrastructure attacks in Nigeria and their associated physical and economic consequences. The methodological framework is designed to support transparent documentation and reproducible description of temporal, sectoral, and volumetric patterns.

### 4.1 Scope and temporal coverage

The geographical scope of the dataset covers Nigeria's six geopolitical zones: North-East, North-West, North-Central, South-East, South-West, and South-South. These zones encompass the country's major oil and gas production areas, extensive crude oil and gas pipelines, export terminals, and high-voltage electricity transmission infrastructure.

The temporal scope spans 2009–2025, including the incident-level attack dataset (2011–2025) and the volume-value loss dataset (2009–2025). The difference in temporal coverage reflects data availability. Incident-level records are consistently available from 2011 onward, whereas annual crude oil loss estimates are reported from 2009. Together, these datasets support analysis of attack frequency across sector and region, as well as description of year-on-year variation in physical losses and their monetary valuation, while accommodating differences in data availability and reporting practices.

### 4.2 Data sources

Two complimentary datasets were compiled due to the absence of a dedicated national database systemically tracking energy infrastructure attacks by non-state actors in Nigeria.

#### 4.2.1 Incident-level attack

The first dataset records individual incidents of attacks on energy infrastructure, including oil and gas facilities and electricity transmission systems. Table 3 highlights a summary of data sources and search strategies used to compile the incident-level attack dataset.

**Table 3:** Summary of data sources and search strategy used to compile the incident-level attack dataset.

Source category	Description	References
Security event database	Nigeria Security Tracker, providing systematically documented records of violent incidents and non-state actor activity.	[[6], [11]]
Peer-reviewed literature	Academic studies reporting historical and contemporary attacks on energy infrastructure in Nigeria.	[6]
Media reports	Verified international and domestic news outlets reporting incidents involving oil and gas facilities and electricity transmission infrastructure.	[6]
Government and industry reports	Publicly available reports from Nigerian regulatory agencies and industry sources documenting energy infrastructure disruptions and losses.	[6]
Search strategy	Keyword-based searches conducted using Google Scholar and media archives, combining actor identifiers (e.g., Boko Haram, Niger Delta militants, bandits), infrastructure terms (e.g., pipeline, flow station, transmission tower), and regional modifiers (e.g., Niger Delta, North-East, North-Central).	[[5], [6]]

#### 4.2.2 Annual volume-value loss dataset

The second dataset documents yearly crude losses resulting from pipeline vandalism, oil theft, and spill events. For 2009–2024, the dataset reports: the estimated volume of crude oil lost (in million barrels) and the corresponding economic loss (in billion USD). These estimates were compiled from industry reports and prior studies [6,[8], [9], [12]]. For 2025, crude oil loss estimates were derived from official data released by the Nigerian Upstream Petroleum Regulatory Commission (NUPRC) [7], which reports losses for the period January-July. These figures were linearly interpolated to obtain an indicative annual estimate of approximately 3.5 million barrels.

### 4.3 Data processing and reproducibility

All data extraction, filtering, classification, and aggregation procedures were implemented using Python scripts provided with the datasets in a GitHub repository https://github.com/Diamonds10/data_in_brief_infradataset_Nigeria. The repository includes raw and cleaned datasets; scripts for keyword-based filtering, date parsing, deduplication, and classification; codebooks documenting variable definitions and assumptions; and validation notes describing data limitations and uncertainty.

These materials are intended to enable transparency, reproducibility, and reuse of the dataset for further academic or industry-driven analysis, risk assessment, and energy systems modelling applications.

#### 4.3.1 Incident classification and screening

Each retrieved incident was manually screened using predefined inclusion criteria. Incidents were included if they involved intentional disruption of oil and gas or electricity transmission systems; were attributed to a non-state actor or recorded as actor unknown; contained sufficient temporal information to assign a year and reporting period; and provided adequate location detail to enable assignment to a geopolitical zone.

Duplicate or near-duplicate records were identified and consolidated through cross-source triangulation based on event date, location, and description. Events failing to meet the inclusion criteria were excluded.

#### 4.3.2 Sectoral and spatial classification

Included records were classified by infrastructure sector (i.e., oil and gas versus power) using a rule-based approach that prioritized explicit infrastructure identifiers (e.g., transmission towers, substations, pipelines) and corroborating textual descriptions in the source material. Spatial classification was conducted by assigning incidents to Nigeria's geopolitical zones based on reported state locations, using a pre-defined state-to-zone concordance in the dataset.

### 4.4 Technical validation

To ensure the quality of datasets, three validation steps were taken. First, all recorded attack incidents were cross-checked and validated across sources. Incidents reported by multiple sources were consolidated into single records with expanded source traceability, while duplicates were identified and removed through matching event date, location, and actor information. Second, spatial accuracy was assessed during the geocoding process. Incident locations were geo-coded using administrative shapefiles.

Records lacking sufficient spatial detail for reliable geocoding were excluded from the spatial dataset. Finally, internal consistency checks were applied to the crude losses and attacks dataset. Reported volumes were of crude oil losses, and associated monetary values were compared across multiple industry and regulatory sources to identify outliers and reconcile discrepancies, ensuring consistency of physical and economic loss records over time.

## Limitations

Several limitations of the dataset should be acknowledged. First, reporting bias may affect the completeness of incident records, as attacks occurring in remote or highly insecure areas may be under-reported in media and secondary sources. In addition, variations in terminology and reporting practices across sources may introduce inconsistencies in incident classification and interpretation. To mitigate this, incidents were triangulated across multiple independent sources wherever possible, prioritizing events corroborated by at least two credible outlets, including regulatory bodies, scholarly databases, non-governmental sources, and established media organizations. Second, geocoding precision of attack locations vary across records, particularly for earlier incidents, with some locations reported at local or regional levels rather than exact coordinates. This was addressed by assigning incidents to geopolitical zones using a predefined state-zone structure and excluding records lacking sufficient spatial detail from analyses requiring precise geolocation. Third, volume-value estimates of crude oil losses rely on secondary sources and simplifying assumptions. Reported figures were cross-checked across industry and regulatory publications, and conservative parameters were applied where discrepancies occurred. These estimates should therefore be interpreted as indicative rather than exact. Finally, actor attribution may be uncertain where responsibility was contested. Such incidents were classified as actor-unknown to maintain consistency and avoid over-assignment.

## Ethics Statement

The authors have read and follow the ethical requirements for publications in Data in Brief and confirming that the current work does not involve human subjects, animal experiments, or any data collected from social media platforms.

## CRediT authorship contribution statement

**Haruna Inuwa:** Writing – review & editing, Writing – original draft, Visualization, Software, Methodology, Formal analysis, Data curation, Conceptualization. **Alycia Leonard:** Writing – review & editing, Supervision, Conceptualization. **Stephanie Hirmer:** Writing – review & editing, Supervision, Project administration, Conceptualization.

## Data Availability

ZenodoMilitia, Boko Haram, Banditry, and Infrastructure Bursts: Impacts on Nigerian Energy Systems (Original data).
